# A Novel Preparation of Nano-Copper Chalcogenide (Cu_2_S)-based Flexible Counter Electrode

**DOI:** 10.1038/s41598-019-48809-7

**Published:** 2019-08-26

**Authors:** Enli Wu, Jingsha Jin, Shaowen Liu, Dan Li, Shufang Gao, Fei Deng, Xuemin Yan, Yan Xiong, Haolin Tang

**Affiliations:** 1 School of Physics and Optoelectronic Engineering, Yangtze University, Jingzhou, 434023 P.R. China; 2 College of Chemistry and Environmental Engineering, Yangtze University, Jingzhou, 434023 P.R. China; 3 State Key Laboratory of Advanced Technology for Materials Synthesis and Processing, Wuhan University of Technology, Wuhan, 430070 P.R. China

**Keywords:** Nanoparticle synthesis, Electronic devices

## Abstract

Copper nanowires (CuNWs) are used to prepare flexible, transparent conducting substrates due to their low cost and ease of fabrication on a large area. A CuNW/polymer composite substrate was prepared and vulcanized to create a novel flexible nano-Cu_2_S/polymer composite substrate. The physical and photovoltaic properties of the substrate can be controlled by tuning the concentration of CuNW dispersion during the preparation of CuNWs and nano Cu_2_S films. The nano-Cu_2_S-based composite substrate was used as an effective flexible counter electrode of a quantum-dot-sensitized solar cell (QDSSC) and resulted in a maximum cell efficiency of 1.01%.

## Introduction

Dye-sensitized solar cells (DSSCs) have a photoelectric conversion efficiency of over 12%, making them a potential candidate for next-generation solar cells^1,2^. Quantum-dot-sensitized solar cells (QDSSCs) use semiconductor quantum dots (QDs) in sensitizers, instead of organic dyes. As a result, QDSSCs exhibit the unique advantages of quantum size effect, multi-exciton effect, large absorption coefficient, and easy matching of energy levels between the electron donor and acceptor materials^3,4^. The structure of a QDSSC is sandwich-like and mainly consists of a photoanode, electrolyte, and counter electrode. The photoanode is mainly composed of a conductive transparent substrate, such as fluorine-doped tin oxide (FTO) glass, with an overlying semiconductor oxide film such as TiO_2_, ZnO that adsorbs a sensitizer, i.e. QDs. A polysulfide electrolyte works as a redox couple. The counter electrode, generally copper chalcogenide (Cu_2_S or CuS), serves as a reduction catalyst^5,6^. CdS/CdSe-cosensitized TiO_2_ is widely studied as a classical co-sensitization system. The CdS QDs adsorbed on the TiO_2_ films show a good effect on the deposition of CdSe QDs, finally forming a classical TiO_2_/CdS/CdSe cascade structure^6,7^.

Copper is the most frequently used metal material for industrial and commercial applications. Recent research has focused on the applications of copper nanowires (CuNWs)^8,9^. A nanowire-based transparent conductive film has the advantages of excellent photovoltaic performance, low preparation cost, and it could be used to prepare flexible devices. CuNWs are being used to prepare flexible transparent conducting substrates because of their low cost and ease of fabrication on a large area^10–12^.

While DSSCs typically use Pt- or Au-coated conducting glass as the counter electrode, QDSSCs usually use a copper-chalcogenide-based counter electrode. This is because the sulfur-containing electrolyte absorbs preferentially and strongly on the Pt or Au surface, leading to surface passivation and decrease in the conductivity of electrodes^13^. Bulk and nanostructured copper chalcogenides are used as the counter electrodes in QDSSCs. The bulk copper chalcogenides are mainly made using brass, and they have the best cell efficiency. However, the cells suffer from mechanical and chemical instability^13–15^. The nanostructured copper-chalcogenide-based counter electrodes are usually fabricated by either synthesizing Cu_2-x_S and then coating it on the conducting glass, or by assembling nano Cu_2_S arrays on the rigid substrate^16^.

In this study, a flexible CuNW-based composite substrate was prepared and then vulcanized to create a flexible nano-Cu_2_S-polymer composite substrate. To the best of our knowledge, this is the first time that a nano Cu_2_S film was fabricated as described, and a flexible nano-Cu_2_S-based counter electrode on a polymer substrate was used in QDSSCs. The physical and photovoltaic properties of the substrate can be controlled by tuning the concentration of CuNW dispersion during the preparation of CuNWs and nano Cu_2_S films. The novel composite substrate functioned well as the flexible counter electrode of a CdS/CdSe QD co-sensitized solar cell.

## Materials and Methods

### Materials

Cadmium nitrate tetrahydrate (Cd(NO_3_)_2_·4H_2_O ≥ 98.0%), sodium sulfide nonahydrate (Na_2_S·9H_2_O ≥ 98.0%), selenium (Se ≥ 99.5%), sodium sulfite (Na_2_SO_3_ ≥ 98.0%), cadmium sulfate hydrate (CdSO_4_·8/3H_2_O ≥ 99.0%), nitrilotriacetic acid (C_6_H_9_NO_6_ ≥ 99.0%), 2, 2-Dimethoxy-2-phenyl-acetophenone (DMPA), Sulfur (S), potassium chloride (KCl ≥ 99.5%), nitrilotriacetic acid and potassium hydroxide (KOH ≥ 85.0%) were purchased from Sigma-Aldrich. lsopropyl alcohol, methanol, ethanol, and acetone were obtained from Sinopharm Chemical Reagent Co., Ltd. (Shanghai, China). Conducting FTO glass and commercial TiO_2_ nanoparticle (P25)-coated FTO were acquired from Yinkou OPV Tech New Energy Co. Ltd. (Yinkou, China). The TiO_2_-coated FTO glass had an effective area of 0.16 cm^2^. CuNWs and PVP ((C_6_H_9_NO)_n_) ≥ 97%) were purchased from Suzhou Tanfeng Tech Co., Ltd. (Suzhou, China). The acrylate monomer ethoxylated (4) bisphenol a dimethacrylate was purchased from Changxing Chemical Co., Ltd. (Zhuhai, China)^17^. All of the chemicals were reagent grade and used without further purification.

### Preparation of CdS/CdSe photoanode

For making CdS QDs, a TiO_2_ film was first dipped into an ethanol solution containing 0.1 M Cd(NO_3_)_2_ for 1 min and rinsed with ethanol. Then, the film was dipped for another 1 min into a 0.1 M Na_2_S methanol solution and rinsed with methanol. This two-step dipping procedure is regarded as one successive ionic layer adsorption and reaction (SILAR) cycle, and the incorporated amount of CdS can be increased by repeating the assembly cycles. A total of 12 SILAR cycles were performed, and then the glass was air dried^6,18^. Next, CdSe was deposited on top of the CdS-coated glass by a chemical bath deposition (CBD) method. The CdSe deposition was achieved by using nitriloacetate as a complex and selenosulfate as an Se source. First, for the Se source, Na_2_SeSO_3_ aqueous solution was freshly prepared by refluxing 0.2 M Se powder in an aqueous solution of 0.5 M Na_2_SO_3_ at 70 °C, for approximately 5 h. Nitrilotriacetic acid and KOH were mixed to prepare K_3_NTA solution. Then, a solution was prepared by mixing 80 mM CdSO_4_, 160 mM K_3_NTA, and 80 mM K_2_SeSO_3_. To promote CdSe QD adsorption, the TiO_2_ electrodes adsorbed with CdS QDs were placed in the solution at room temperature in the dark for 4 hours^6^.

### Preparation of CuNW-polymer composite substrate

Prior to the procedure, all of the glass substrates used in the preparation process were cleaned using detergent, deionized water, acetone, and isopropyl alcohol, under ultrasonication. CuNWs were dispersed in isopropanol with 1% (mass ratio) of PVP as agent^9^. Here, PVP was used as a surface modified and stabilized reagent to prevent the nanowires from coalescing. The dispersion was treated under ultrasonication to obtain a good uniformity.

A CuNW film can be prepared by different methods, such as spin coating, Mayer rod coating, and spray coating^10,19,20^. In this study, to fabricate a relatively thick film of CuNWs, a dispersion of CuNWs in isopropanol was drop cast on the glass substrate at room temperature. After the required thickness of CuNWs was drop cast, the glass substrate was air dried. Afterwards, the acrylate monomer with 1 wt% DMPA as a polymerization initiator was coated on the CuNW coating. The coating was then cured under an ultraviolet curing conveyor for approximately 90 s and peeled off as a transparent flexible composite^17^.

### CuNW vulcanization

In a water and methanol (1:1 volume) solution, 0.1 M Na_2_S, 0.1 M S, and 0.2 M KCl were added to prepare a solution. The previously prepared CuNW-based flexible composite was placed in this solution for 3–5 min to generate nano Cu_2_S on the surface of the composite.

### QDSSC device fabrication and characterization

The sensitized TiO_2_ film was used as a photoanode and the nano-Cu_2_S-based composite substrate was used as the counter electrode. The electrolyte, which consisted of 0.1 M Na_2_S, 0.1 M S and 0.2 M KCl in a water and methanol (1:1 volume) solution, was injected between the photoanode and counter electrode by siphonic action.

The photovoltaic performances [short circuit current (*J*sc), open circuit voltage (*V*oc), fill factor (*FF*), and power conversion efficiency (*η*)] of the cell were measured. The current density-voltage (*J-V*) characteristics of the cells were measured using a Keithley 2450 source meter for a light intensity of 100 mW/cm^2^ from a Xenon lamp (300 W; Nbet, HSX-F300). The microstructure of the CuNWs was analyzed with a field emission scanning electronic microscope (SEM; JEOL, JSM7100F). Electrochemical impedance spectroscopy (EIS) measurements were obtained using an electrochemical workstation (CorrTest, CS 350 H). Elemental analysis was performed with an energy dispersive spectrometer (EDS, Oxford X-MAX). X-ray diffraction (XRD) patterns were recorded by an X-ray diffractometer (Empyrean, PANalytical).

## Results and Discussion

**Figure 1:**
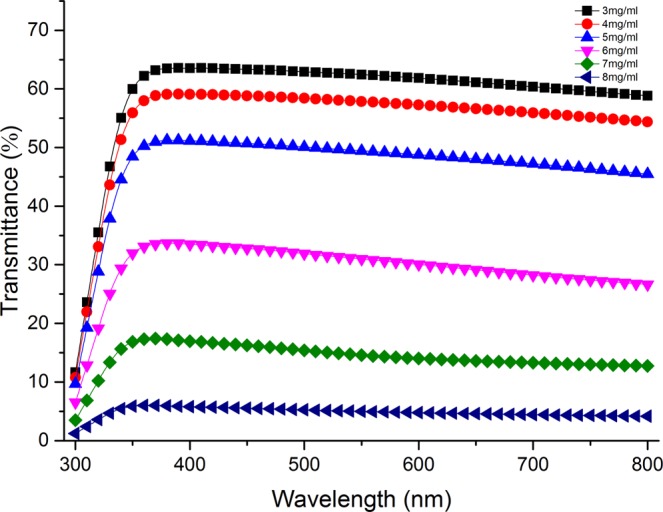
Transmittance of CuNW-based flexible composite substrates prepared with dispersion of different CuNW concentrations.

Figure 1 shows the transmittance of CuNW-based flexible composite substrates prepared with dispersion of different CuNW concentrations. The transmittance of the film reflects the thickness and compactness of the film. This gives an indication of the quality of the film. For the substrates prepared with CuNW dispersion concentrations less than 5 mg/ml, the transmittance in the wavelength range of 350–800 nm is higher than 50%. As the CuNW concentration of the dispersion increases from 3 mg/ml to 8 mg/ml, the transmittance of the substrate gradually decreases. This decrease happens because the distribution density of the nanowires grows with the dispersion concentration.

**Figure 2:**
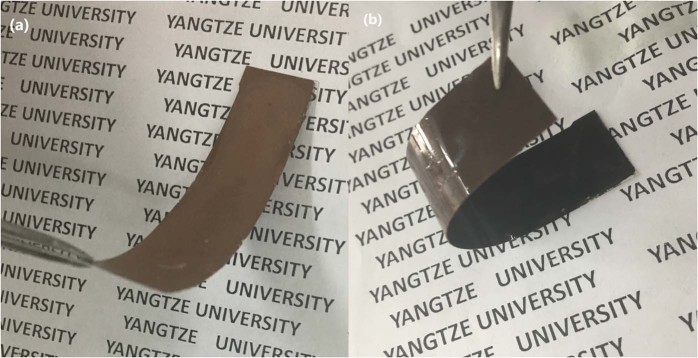
CuNW-based flexible composite substrate (**a**) and vulcanized substrate (**b**).

Figure 2 shows a CuNW-based composite substrate (Fig. 2a) and a vulcanized substrate (Fig. 2b). Both substrates have good flexibility. After CuNW vulcanization, the color of the substrate turned from bronze to black, which is the typical color of Cu_2_S. The average thicknesses of the CuNW layer and composite substrate were 4 μm and 44 μm, respectively.

**Figure 3:**
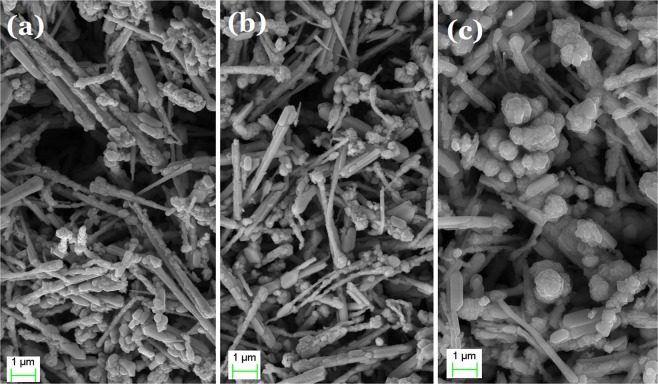
Scanning electronic microscope (SEM) images of the surface of CuNW-based flexible composite substrates, prepared with dispersion of CuNW concentrations of (**a**) 5 mg/ml, (**b**) 7 mg/ml, and (**c**) 8 mg/ml.

SEM images of the surface of a CuNW-based composite substrate are shown in Fig. 3. The samples were prepared with dispersion of CuNW concentrations of 5 mg/ml, 7 mg/ml, and 8 mg/ml, respectively. CuNWs are evenly distributed on the substrate. As the CuNW concentration is increased, the composite substrate is coated with denser CuNWs.

**Figure 4:**
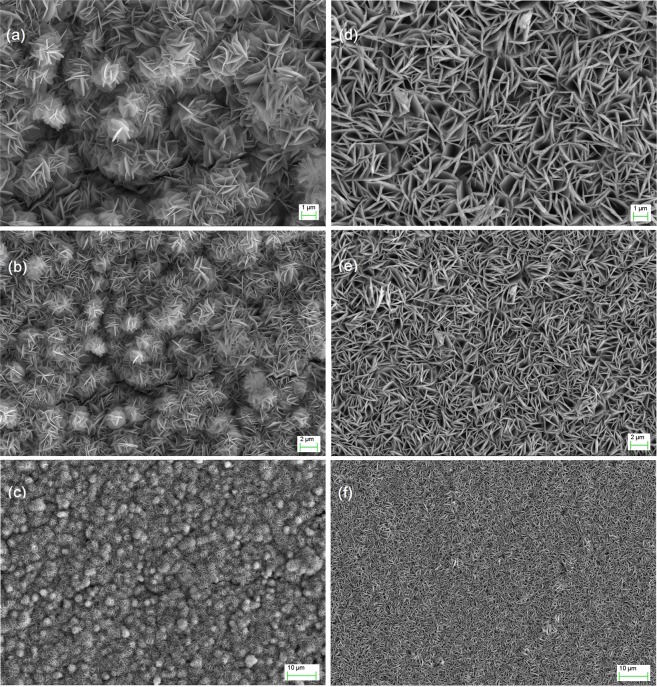
SEM images of the surface of nano-Cu_2_S-based composite substrate prepared with dispersion of CuNW concentrations of 5 mg/ml (**a**–**c**) and 7 mg/ml (**d**–**f**).

The CuNW-based composite substrates that were prepared with CuNW concentrations of 5 mg/ml and 7 mg/ml were sulfided to produce nano Cu_2_S. The SEM images of the surface of these samples are shown in Fig. 4. The Cu_2_S flakes are closely arranged. Compared to nano Cu_2_S fabricated with lower CuNW dispersion concentration (5 mg/ml), the nano Cu_2_S prepared with a higher CuNW dispersion concentration (7 mg/ml) are more densely distributed. The morphology of the nano Cu_2_S is similar to the morphology found in literature^13,21^.

To further confirm the vulcanization of CuNWs, EDS analysis was conducted to investigate the elemental compositions of non-vulcanized and vulcanized CuNWs on the flexible substrates. Samples of CuNW-based composite substrates, which were fabricated with dispersion of different CuNW concentrations were analyzed. The details of the EDS analysis are listed in Table 1. The samples were primarily composed of C, O, and Cu. The Cu atomic percentage gradually increased as the CuNW dispersion concentration increases. The vulcanized samples were primarily composed of C, O, S, Cu, and Cu_2_S. After vulcanization, the atomic percentage of Cu in the sample decreased. This decrease may be caused by the loss of CuNWs during the vulcanization in the solution. The ratio of S atoms to Cu atoms are similar for CuNW concentrations between 5 mg/ml to 8 mg/ml.

**Table 1:** Element distribution of CuNW-based composite substrate and vulcanized substrate, as seen using EDS analysis.

	Dispersion concentration	3 mg/ml	4 mg/ml	5 mg/ml	7 mg/ml	8 mg/ml
	Atomic percentage					
CuNW	C-K	72.81	55.86	57.37	54.21	49.05
	O-K	13.43	19.56	16.52	9.89	8.25
	Cu-L	14.19	24.56	25.98	35.53	42.48
Vulcanized CuNW	C-K	—	—	54.86	54.64	50.29
	O-K	—	—	13.42	13.73	14.07
	S-K	—	—	7.47	7.56	7.70
	Cu-L	—	—	24.19	23.96	27.68

**Figure 5:**
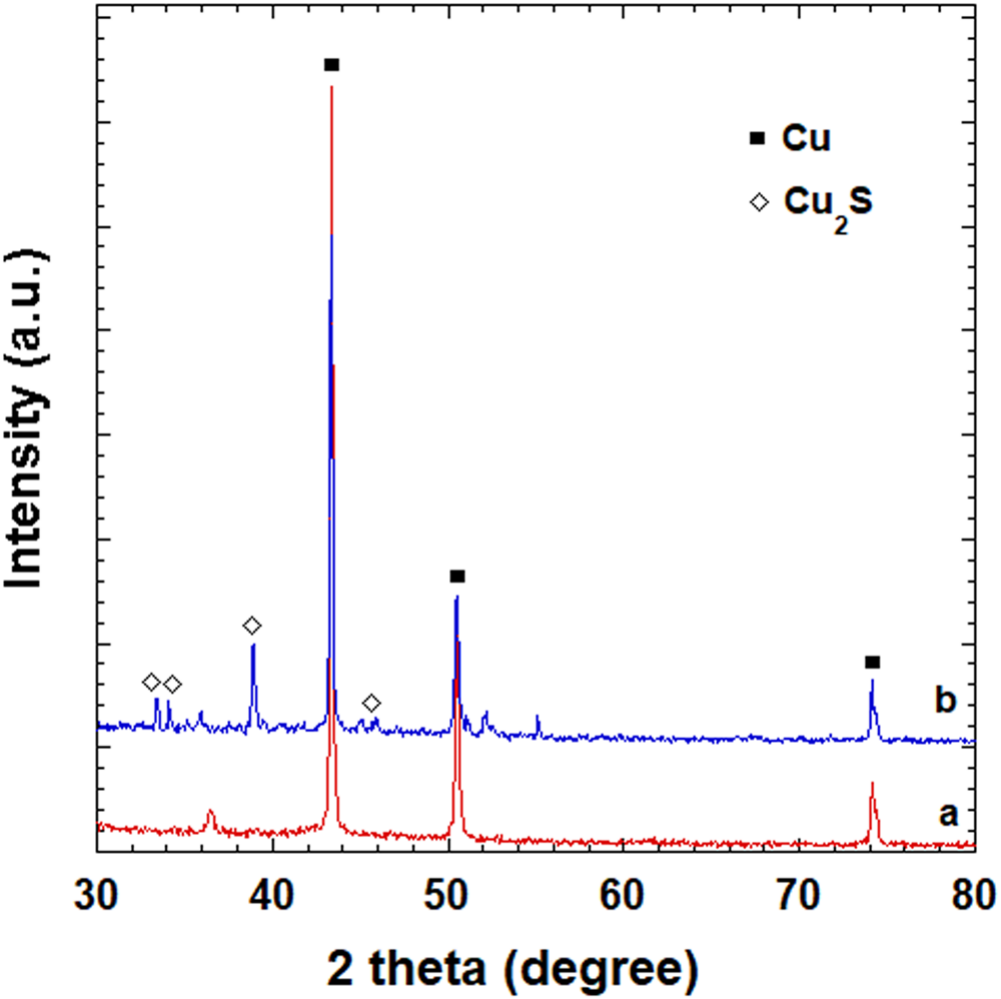
X-ray diffraction patterns of CuNW-based flexible composite substrate (**a**) and vulcanized substrate (**b**), prepared with dispersion of CuNW concentrations of 8 mg/ml.

The vulcanization of CuNWs was also confirmed by the XRD patterns presented in Fig. 5. The diffraction peaks of the CuNW/polymer composite matched well with known Cu peaks (JCPDS 04-0836). After vulcanization, new peaks corresponding to Cu_2_S (JCPDS 33-0490) were observed.

**Figure 6:**
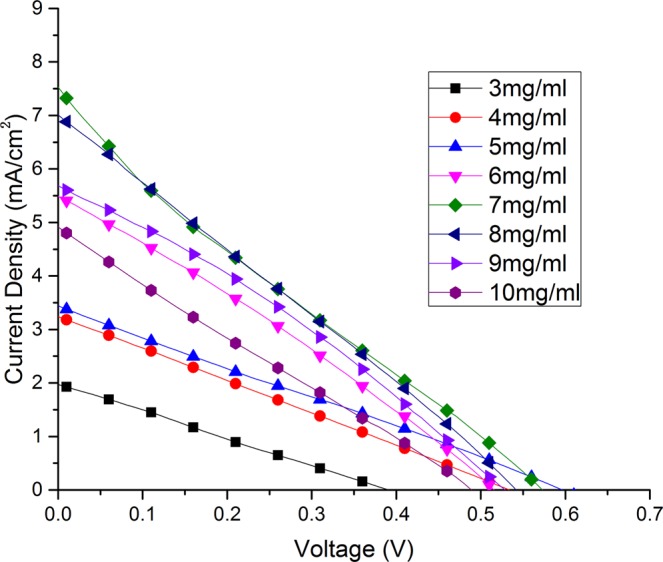
*J-V* characterization of the solar cells, where *J* is the current density (mA/cm^2^), and *V* is the voltage.

Figure 6 shows the *J*–*V* characteristics of the solar cells using a nano-Cu_2_S-based composite substrate as counter electrode. Table 2 shows the key photovoltaic parameters (*J*sc, *V*oc, *FF*, and maximum total energy conversion efficiency, *η*) of the devices. As the concentrations of CuNWs in the dispersion increases from 3 to 8 mg/ml, the cell efficiency of the corresponding QDSSCs first increases. A maximum efficiency of 1.01% is achieved when the CuNW dispersion concentration is 8 mg/ml. After this, the efficiency decreases as the CuNW concentration is increased to 9 mg/ml and 10 mg/ml. The cell efficiency is 0.8% or more when the dispersion concentration of CuNWs is between 6 mg/ml and 9 mg/ml. This increased energy conversion efficiency can be mainly attributed to an increase in the photocurrent. The open circuit voltage of the QDSSCs with CuNW concentrations between 4 mg/ml and 9 mg/ml is in the range of 0.52 V to 0.6 V.

**Table 2:** Photovoltaic parameters of the solar cells.

Dispersion concentration (mg/ml)	V_oc_ (V)	J_sc_ (mA/cm^2^)	η_max_ (%)	FF
3	0.34	1.88	0.19	0.29
4	0.54	3.23	0.43	0.25
5	0.60	3.42	0.53	0.26
6	0.54	5.50	0.80	0.26
7	0.57	7.79	0.99	0.22
8	0.57	6.89	1.01	0.25
9	0.52	5.66	0.90	0.30
10	0.48	4.92	0.59	0.24

As the CuNW concentration in the dispersion increases, the as-prepared composite substrate and the corresponding vulcanized substrate obtains denser CuNWs or nano Cu_2_S on the substrate. Thus, the efficiency of the QDSSCs using nano Cu_2_S counter electrode increased until the saturation point is reached at about 7 mg/ml–8 mg/ml.

It should be noted that, during the fabrication of a CuNW/polymer composite, as the CuNW concentration was increased, the density of the drop-cast CuNW film on the glass substrate increased. It took less time for a CuNW film made with a higher CuNW concentration to air dry. In addition, CuNWs made with a higher CuNW concentration dispersion are expected to aggregate more easily. As the CuNW concentration was increased to more than 9 mg/ml, it was difficult to completely transfer CuNWs from the glass substrate to the acrylate polymer film; after the CuNW/polymer film was peeled off from the glass substrate, some CuNWs were still left on the glass substrate. These factors lead to a low efficiency of QDSSCs when the CuNW dispersion concentration is 10 mg/ml.

**Figure 7:**
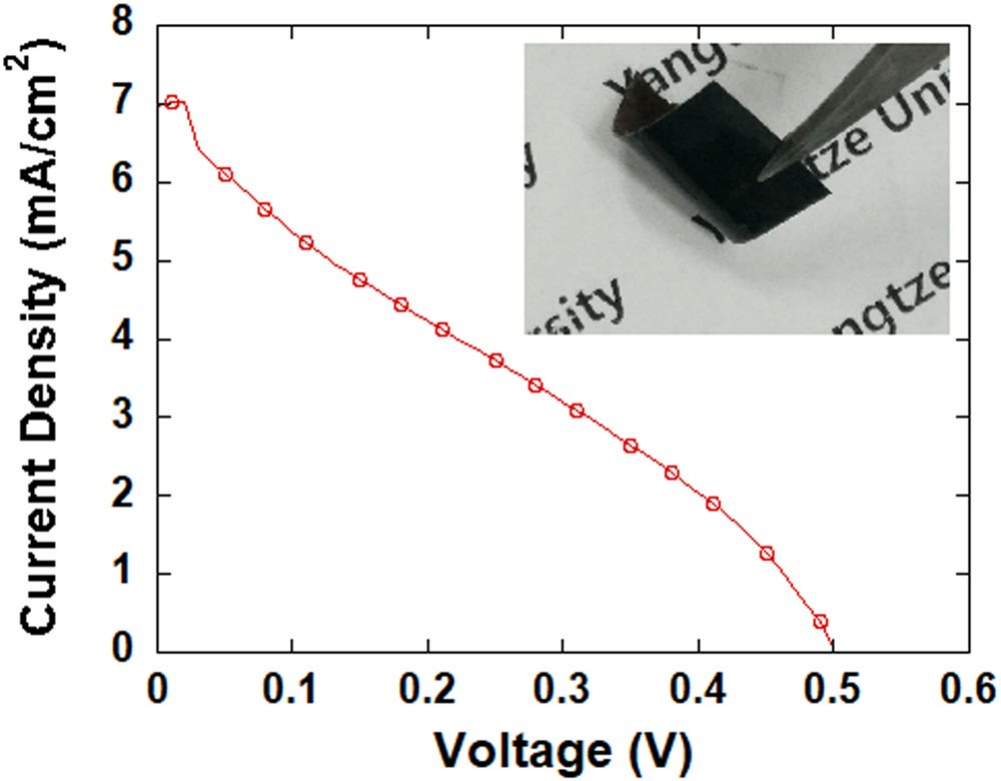
*J-V* characterization of the QDSSC using a nano-Cu_2_S-based composite substrate that was bent 200 times, where *J* is the current density (mA/cm^2^), and *V* is the voltage. Inset: nano-Cu_2_S-based counter electrode after the photovoltaic test.

To evaluate the flexibility of nano-Cu_2_S-based counter electrodes, they were subjected to a bending test. The nano Cu_2_S counter electrode was bent along a curve of radius 0.7 cm, which corresponds to a curvature of 143 m^−1^. After repeatedly bending the electrode 200 times, a tiny decrease in cell performance was observed. The cell efficiency was acceptable at 0.96%. The *J*sc, *V*oc and *FF* were 0.50 V, 7.08 mA/cm^2^, and 0.27, respectively. Figure 7 shows the *J*–*V* characteristics of the solar cells using a nano-Cu_2_S-based composite substrate that was bent 200 times. No fall-off or breakage was observed even after the photovoltaic test (Fig. 7, inset). While bulk copper chalcogenide electrodes are usually prepared by immersing a polished brass in a (poly)sulfide solution^14,15^, when Cu_2_S coated brass is employed as the counter electrode of QDSSCs, the Cu_2_S on the surface exfoliates easily. Thus, the novel flexible nano-Cu_2_S-based counter electrode has better flexibility and mechanical stability than bulk copper chalcogenide electrodes.

**Figure 8:**
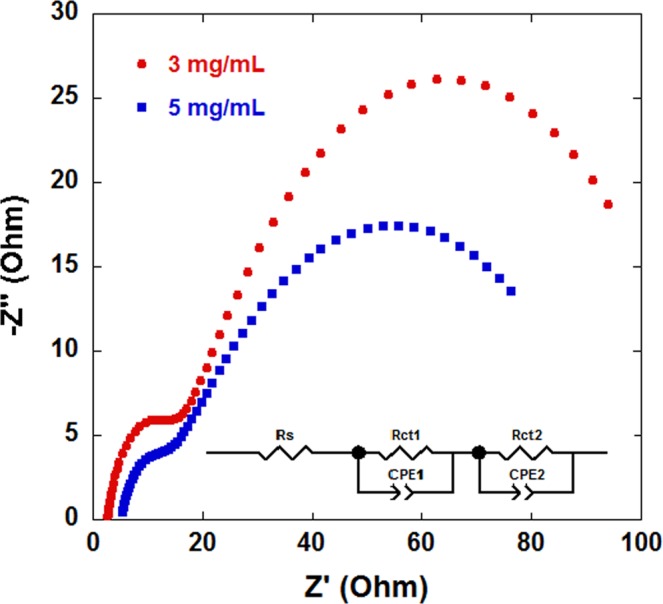
Nyquist plots of the QDSSCs under 100 mW/cm^−2^ illumination and frequency from 0.1 Hz to 500 KHz at room temperature. Inset: equivalent circuit model of the QDSSCs.

The interface characteristics of the QDSSCs were studied by the EIS method. Figure 8 shows the Nyquist plots of QDSSCs under illumination of 100 mWcm^−2^. For CuNW dispersion concentrations of 3 mg/ml and 5 mg/ml, two semicircles are observed in the Nyquist curves for both CuNW concentrations. The smaller semicircle appears at high frequencies and represents the redox impedance of the counter electrode interface. The lager semicircle appears at middle frequencies and represents the impedance at the photoanode/dye (QDs)/electrolyte interface. The semicircles can be represented by an equivalent circuit, as shown in Fig. 8 (inset)^22,23^. The equivalent circuit is composed of a series resistance *R*_s_, transfer resistances *R*_ct1_ and *R*_ct2_, and chemical capacitances *CPE*_1_ and *CPE*_2_. *R*_ct1_ represents the charge transfer resistance at the interface of the electrolyte and counter electrode. *R*_ct2_ represents the charge transfer resistance at the interface of TiO_2_/QD/electrolyte. *CPE*_1_ and *CPE*_2_ are constant phase elements of the capacitances corresponding to *R*_ct1_ and *R*_ct2_, respectively. Table 3 shows that a substrate prepared with a CuNW dispersion concentration of 5 mg/ml has a smaller *R*_ct1_ value than that of a dispersion concentration of 3 mg/ml. This trend indicates that the carriers are easier to transfer at an electrode with denser nano Cu_2_S.

**Table 3:** Photovoltaic properties obtained by fitting the impedance spectra of QDSSCs using the equivalent circuit shown in Fig. 8.

Dispersion concentration	R_s_ (Ω)	R_ct1_ (Ω)	R_ct2_ (Ω)
3 mg/ml	2.620	7.685	43.61
5 mg/ml	3.608	5.455	86.48

The Cu_2_S-polymer composite used in this study is easy to fabricate. In addition, once CuNWs have been synthesized, they can be directly used to prepare a nano Cu_2_S film. The density and morphology of the distributed nano Cu_2_S on the polymer substrate is controllable by tuning the CuNW dispersion concentration. This convenient and simple method can also be generalized to the fabrication of similar flexible electrodes. The performance of the as-prepared counter electrode depends a lot on the quality of the CuNWs and the corresponding nano Cu_2_S film. The morphology and dispersity of CuNWs affect the electrical conductivity, uniformity, and vulcanization of the CuNW-polymer composite substrate, and consequently of the Cu_2_S-polymer composite counter electrode. The efficiency of QDSSCs is expected to increase with the improvement of CuNWs, and we are exploring this.

## Conclusion

CuNW films were prepared on glass substrates by CuNW dispersion. A flexible CuNW-polymer composite substrate was then prepared by coating an acrylate monomer onto the CuNW coated glass and then curing under ultraviolet light. After vulcanization treatment, the CuNWs turned into nano Cu_2_S and a novel flexible nano-Cu_2_S-based composite substrate was obtained. The nano-Cu_2_S based substrate was used as a counter electrode of QDSSCs. A maximum cell efficiency of 1.01% was observed. The effects of the concentration of CuNW dispersion on the physical and photovoltaic properties of the CuNW film, nano Cu_2_S film, and QDSSCs were investigated by absorption spectroscopy, energy dispersive spectrometry, SEM, XRD, *J-V* characteristics, and EIS. If the CuNW in the dispersion is thick enough, the as-prepared counter electrode would achieve a denser Cu_2_S film and a lower charge transfer resistance at the interface of  the counter electrode and electrolyte.
